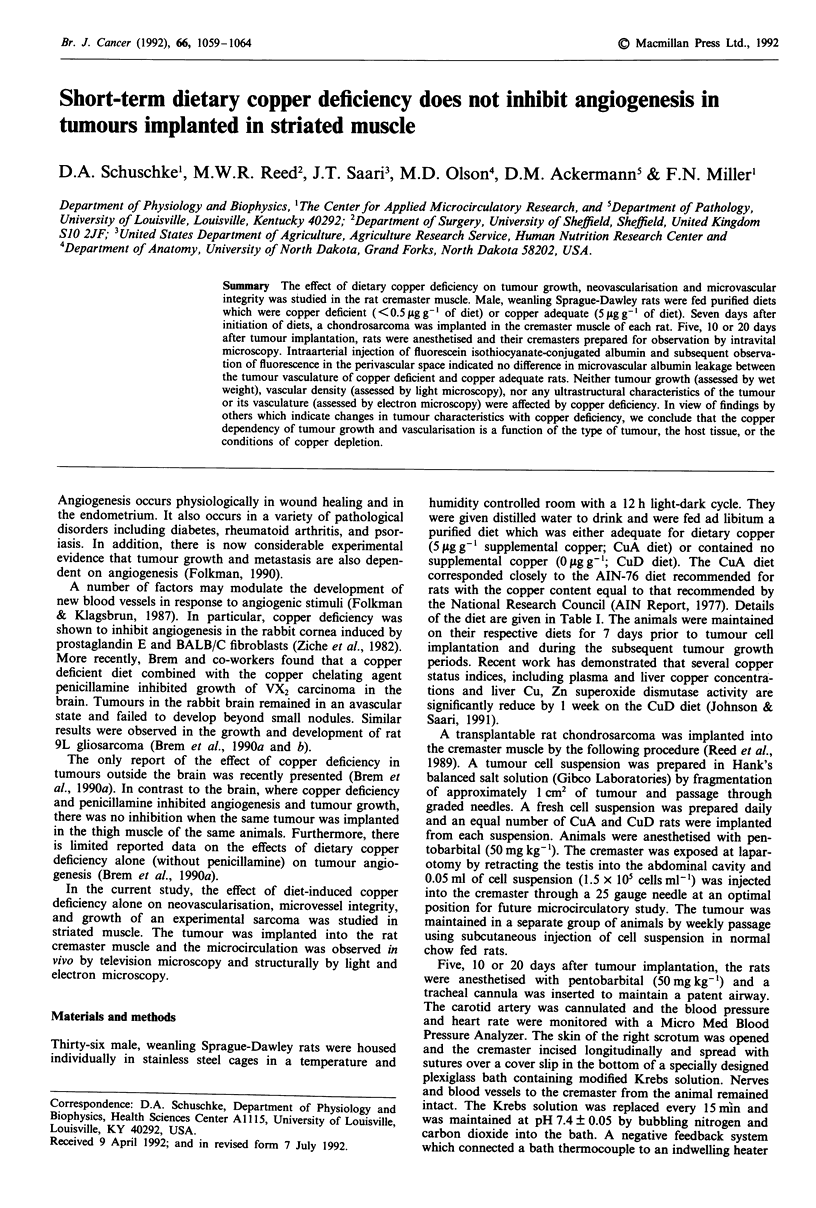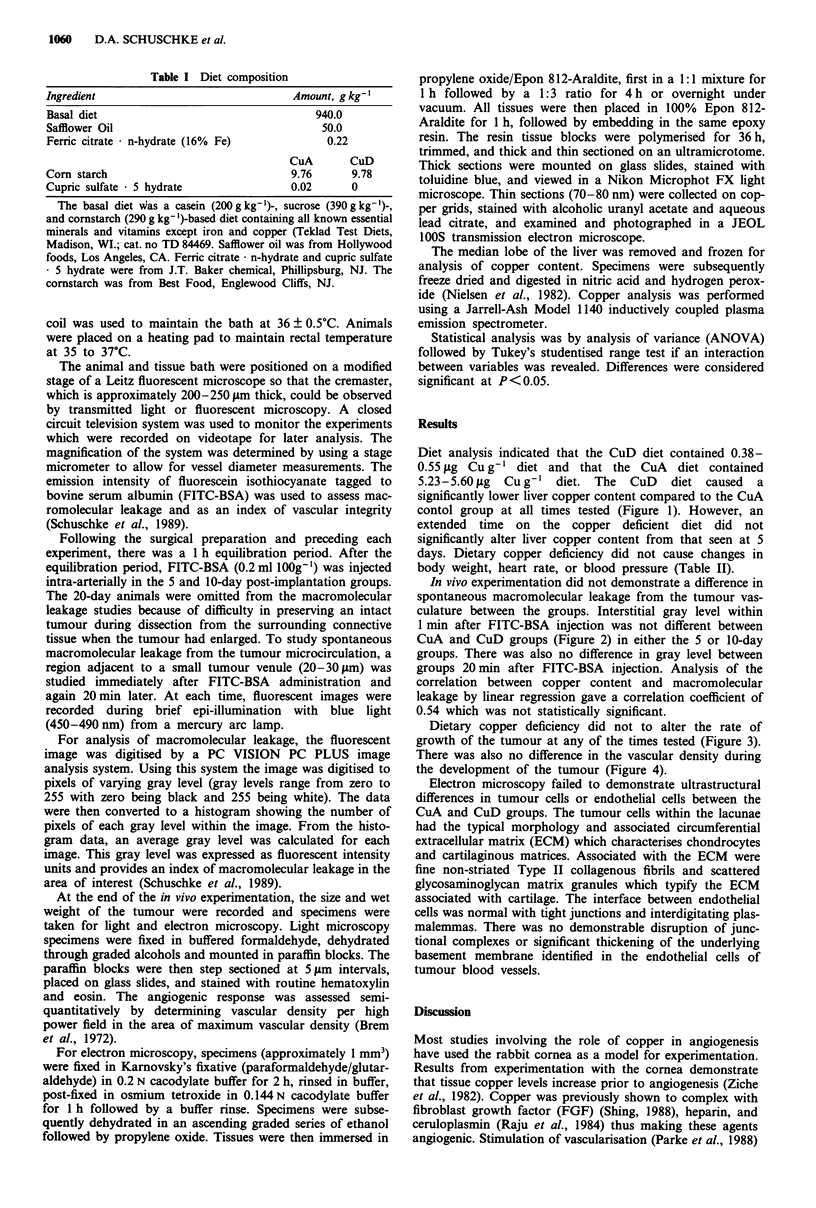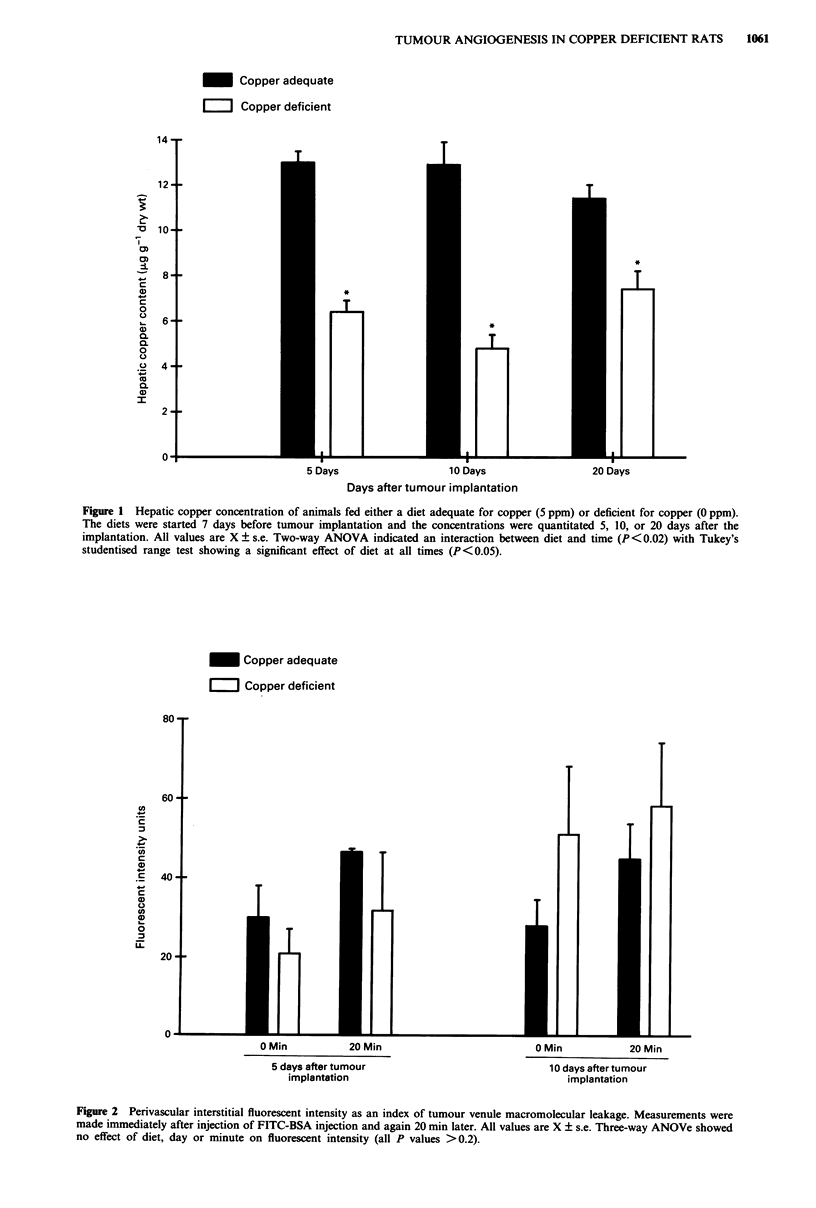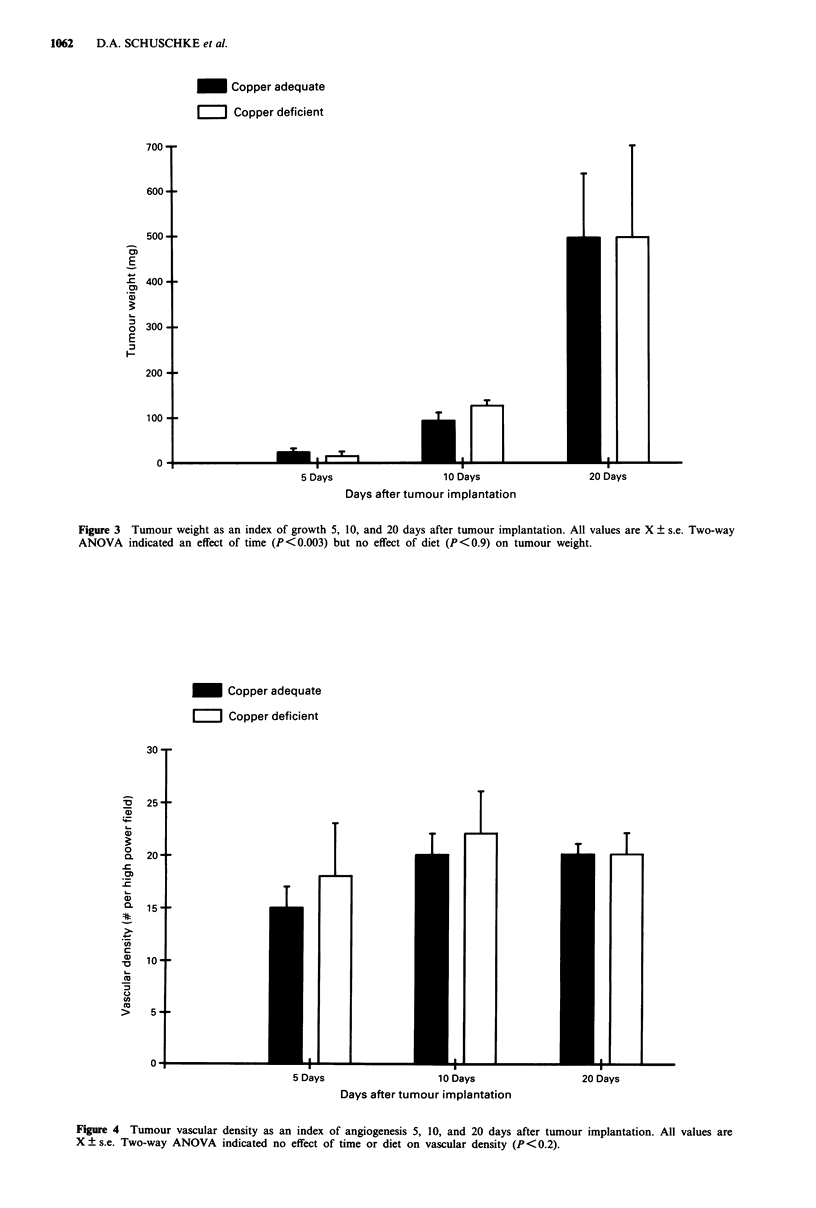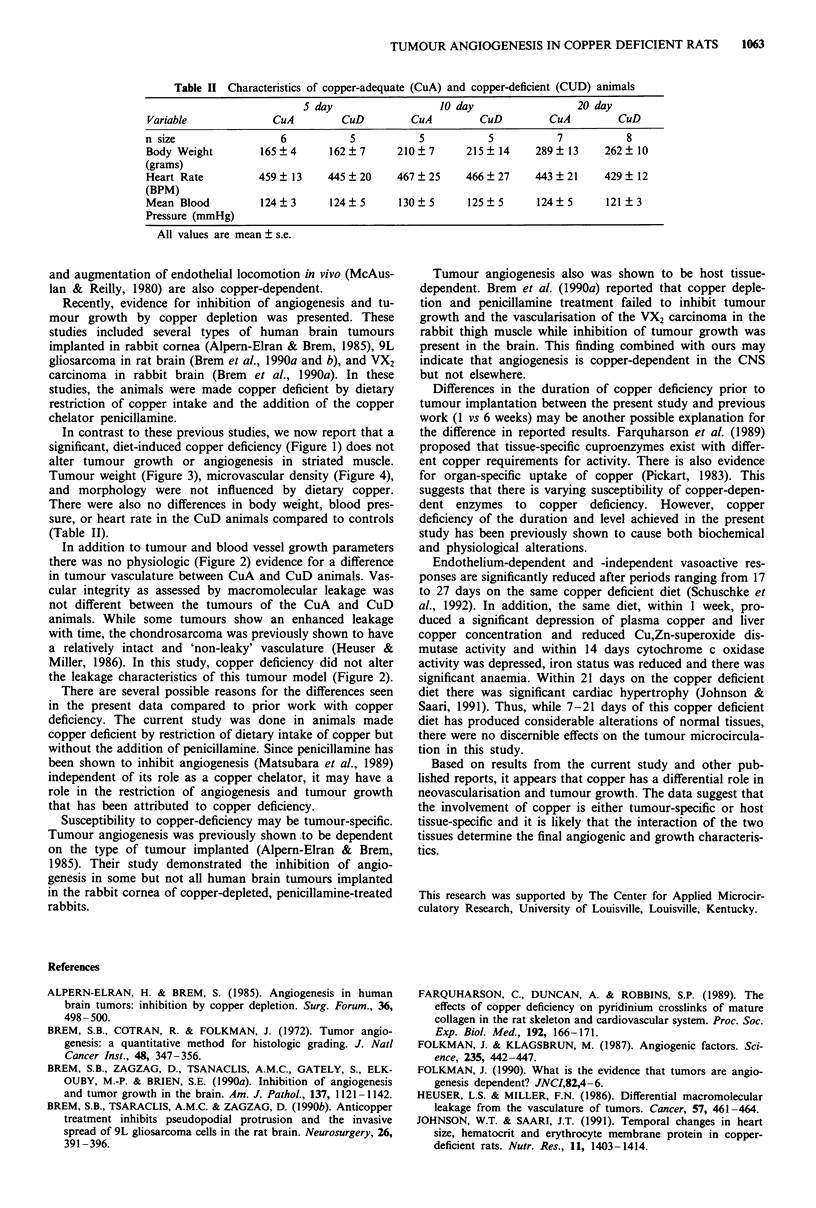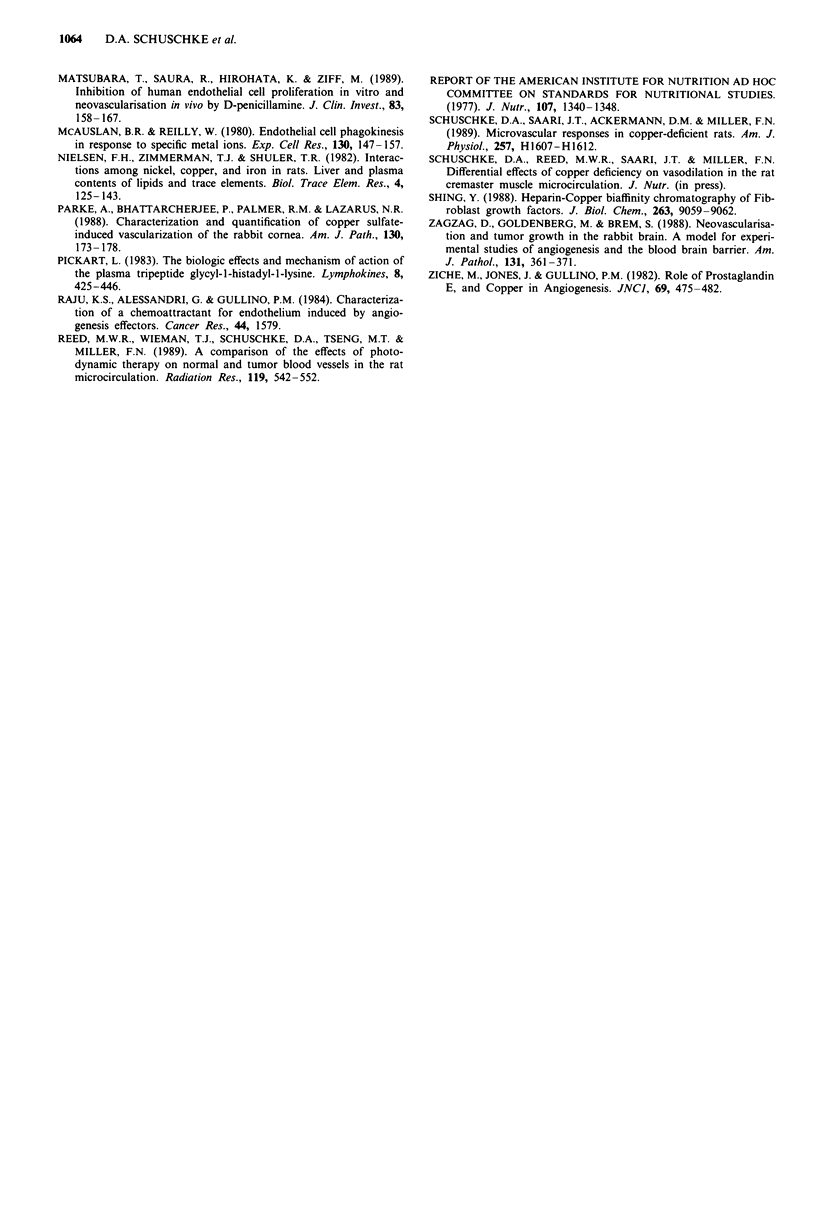# Short-term dietary copper deficiency does not inhibit angiogenesis in tumours implanted in striated muscle.

**DOI:** 10.1038/bjc.1992.410

**Published:** 1992-12

**Authors:** D. A. Schuschke, M. W. Reed, J. T. Saari, M. D. Olson, D. M. Ackermann, F. N. Miller

**Affiliations:** Department of Physiology and Biophysics, University of Louisville, Kentucky 40292.

## Abstract

The effect of dietary copper deficiency on tumour growth, neovascularisation and microvascular integrity was studied in the rat cremaster muscle. Male, weanling Sprague-Dawley rats were fed purified diets which were copper deficient (< 0.5 micrograms g-1 of diet) or copper adequate (5 micrograms g-1 of diet). Seven days after initiation of diets, a chondrosarcoma was implanted in the cremaster muscle of each rat. Five, 10 or 20 days after tumour implantation, rats were anesthetised and their cremasters prepared for observation by intravital microscopy. Intraarterial injection of fluorescein isothiocyanate-conjugated albumin and subsequent observation of fluorescence in the perivascular space indicated no difference in microvascular albumin leakage between the tumour vasculature of copper deficient and copper adequate rats. Neither tumour growth (assessed by wet weight), vascular density (assessed by light microscopy), nor any ultrastructural characteristics of the tumour or its vasculature (assessed by electron microscopy) were affected by copper deficiency. In view of findings by others which indicate changes in tumour characteristics with copper deficiency, we conclude that the copper dependency of tumour growth and vascularisation is a function of the type of tumour, the host tissue, or the conditions of copper depletion.


*Br. J. Cancer* (1992), **66**, 1059–1064 © Macmillan Press Ltd., 1992

### Short-term dietary copper deficiency does not inhibit angiogenesis in tumours implanted in striated muscle

D.A. Schuschke[1], M.W.R. Reed[2], J.T. Saari[3], M.D. Olson[4], D.M. Ackermann[5] & F.N. Miller[1]

*Department of Physiology and Biophysics, [1]The Center for Applied Microcirculatory Research, and [5]Department of Pathology, University of Louisville, Louisville, Kentucky 40292; [2]Department of Surgery, University of Sheffield, Sheffield, United Kingdom S10 2JF; [3]United States Department of Agriculture, Agriculture Research Service, Human Nutrition Research Center and [4]Department of Anatomy, University of North Dakota, Grand Forks, North Dakota 58202, USA.*

**Summary** The effect of dietary copper deficiency on tumour growth, neovascularisation and microvascular integrity was studied in the rat cremaster muscle. Male, weanling Sprague-Dawley rats were fed purified diets which were copper deficient ($<0.5\,\mu g\,g^{-1}$ of diet) or copper adequate ($5\,\mu g\,g^{-1}$ of diet). Seven days after initiation of diets, a chondrosarcoma was implanted in the cremaster muscle of each rat. Five, 10 or 20 days after tumour implantation, rats were anesthetised and their cremasters prepared for observation by intravital microscopy. Intraarterial injection of fluorescein isothiocyanate-conjugated albumin and subsequent observation of fluorescence in the perivascular space indicated no difference in microvascular albumin leakage between the tumour vasculature of copper deficient and copper adequate rats. Neither tumour growth (assessed by wet weight), vascular density (assessed by light microscopy), nor any ultrastructural characteristics of the tumour or its vasculature (assessed by electron microscopy) were affected by copper deficiency. In view of findings by others which indicate changes in tumour characteristics with copper deficiency, we conclude that the copper dependency of tumour growth and vascularisation is a function of the type of tumour, the host tissue, or the conditions of copper depletion.

Angiogenesis occurs physiologically in wound healing and in the endometrium. It also occurs in a variety of pathological disorders including diabetes, rheumatoid arthritis, and psoriasis. In addition, there is now considerable experimental evidence that tumour growth and metastasis are also dependent on angiogenesis (Folkman, 1990).

A number of factors may modulate the development of new blood vessels in response to angiogenic stimuli (Folkman & Klagsbrun, 1987). In particular, copper deficiency was shown to inhibit angiogenesis in the rabbit cornea induced by prostaglandin E and BALB/C fibroblasts (Ziche *et al.*, 1982). More recently, Brem and co-workers found that a copper deficient diet combined with the copper chelating agent penicillamine inhibited growth of $VX_2$ carcinoma in the brain. Tumours in the rabbit brain remained in an avascular state and failed to develop beyond small nodules. Similar results were observed in the growth and development of rat 9L gliosarcoma (Brem *et al.*, 1990*a* and *b*).

The only report of the effect of copper deficiency in tumours outside the brain was recently presented (Brem *et al.*, 1990*a*). In contrast to the brain, where copper deficiency and penicillamine inhibited angiogenesis and tumour growth, there was no inhibition when the same tumour was implanted in the thigh muscle of the same animals. Furthermore, there is limited reported data on the effects of dietary copper deficiency alone (without penicillamine) on tumour angiogenesis (Brem *et al.*, 1990*a*).

In the current study, the effect of diet-induced copper deficiency alone on neovascularisation, microvessel integrity, and growth of an experimental sarcoma was studied in striated muscle. The tumour was implanted into the rat cremaster muscle and the microcirculation was observed *in vivo* by television microscopy and structurally by light and electron microscopy.

### Materials and methods

Thirty-six male, weanling Sprague-Dawley rats were housed individually in stainless steel cages in a temperature and humidity controlled room with a 12 h light-dark cycle. They were given distilled water to drink and were fed ad libitum a purified diet which was either adequate for dietary copper ($5\,\mu g\,g^{-1}$ supplemental copper; CuA diet) or contained no supplemental copper ($0\,\mu g\,g^{-1}$; CuD diet). The CuA diet corresponded closely to the AIN-76 diet recommended for rats with the copper content equal to that recommended by the National Research Council (AIN Report, 1977). Details of the diet are given in Table I. The animals were maintained on their respective diets for 7 days prior to tumour cell implantation and during the subsequent tumour growth periods. Recent work has demonstrated that several copper status indices, including plasma and liver copper concentrations and liver Cu, Zn superoxide dismutase activity are significantly reduce by 1 week on the CuD diet (Johnson & Saari, 1991).

A transplantable rat chondrosarcoma was implanted into the cremaster muscle by the following procedure (Reed *et al.*, 1989). A tumour cell suspension was prepared in Hank's balanced salt solution (Gibco Laboratories) by fragmentation of approximately 1 $cm^2$ of tumour and passage through graded needles. A fresh cell suspension was prepared daily and an equal number of CuA and CuD rats were implanted from each suspension. Animals were anesthetised with pentobarbital ($50\,mg\,kg^{-1}$). The cremaster was exposed at laparotomy by retracting the testis into the abdominal cavity and 0.05 ml of cell suspension ($1.5 \times 10^5$ cells $ml^{-1}$) was injected into the cremaster through a 25 gauge needle at an optimal position for future microcirculatory study. The tumour was maintained in a separate group of animals by weekly passage using subcutaneous injection of cell suspension in normal chow fed rats.

Five, 10 or 20 days after tumour implantation, the rats were anesthetised with pentobarbital ($50\,mg\,kg^{-1}$) and a tracheal cannula was inserted to maintain a patent airway. The carotid artery was cannulated and the blood pressure and heart rate were monitored with a Micro Med Blood Pressure Analyzer. The skin of the right scrotum was opened and the cremaster incised longitudinally and spread with sutures over a cover slip in the bottom of a specially designed plexiglass bath containing modified Krebs solution. Nerves and blood vessels to the cremaster from the animal remained intact. The Krebs solution was replaced every 15 min and was maintained at pH $7.4 \pm 0.05$ by bubbling nitrogen and carbon dioxide into the bath. A negative feedback system which connected a bath thermocouple to an indwelling heater

Correspondence: D.A. Schuschke, Department of Physiology and Biophysics, Health Sciences Center A1115, University of Louisville, Louisville, KY 40292, USA.
Received 9 April 1992; and in revised form 7 July 1992.

Table I Diet composition

| *Ingredient* | *Amount, g kg⁻¹* | |
|---|---|---|
| Basal diet | 940.0 | |
| Safflower Oil | 50.0 | |
| Ferric citrate · n-hydrate (16% Fe) | 0.22 | |
| | CuA | CuD |
| Corn starch | 9.76 | 9.78 |
| Cupric sulfate · 5 hydrate | 0.02 | 0 |

The basal diet was a casein (200 g $kg^{-1}$)-, sucrose (390 g $kg^{-1}$)-, and cornstarch (290 g $kg^{-1}$)-based diet containing all known essential minerals and vitamins except iron and copper (Teklad Test Diets, Madison, WI.; cat. no TD 84469. Safflower oil was from Hollywood foods, Los Angeles, CA. Ferric citrate · n-hydrate and cupric sulfate · 5 hydrate were from J.T. Baker chemical, Phillipsburg, NJ. The cornstarch was from Best Food, Englewood Cliffs, NJ.

coil was used to maintain the bath at 36 ± 0.5°C. Animals were placed on a heating pad to maintain rectal temperature at 35 to 37°C.

The animal and tissue bath were positioned on a modified stage of a Leitz fluorescent microscope so that the cremaster, which is approximately 200–250 μm thick, could be observed by transmitted light or fluorescent microscopy. A closed circuit television system was used to monitor the experiments which were recorded on videotape for later analysis. The magnification of the system was determined by using a stage micrometer to allow for vessel diameter measurements. The emission intensity of fluorescein isothiocyanate tagged to bovine serum albumin (FITC-BSA) was used to assess macromolecular leakage and as an index of vascular integrity (Schuschke *et al.*, 1989).

Following the surgical preparation and preceding each experiment, there was a 1 h equilibration period. After the equilibration period, FITC-BSA (0.2 ml $100g^{-1}$) was injected intra-arterially in the 5 and 10-day post-implantation groups. The 20-day animals were omitted from the macromolecular leakage studies because of difficulty in preserving an intact tumour during dissection from the surrounding connective tissue when the tumour had enlarged. To study spontaneous macromolecular leakage from the tumour microcirculation, a region adjacent to a small tumour venule (20–30 μm) was studied immediately after FITC-BSA administration and again 20 min later. At each time, fluorescent images were recorded during brief epi-illumination with blue light (450–490 nm) from a mercury arc lamp.

For analysis of macromolecular leakage, the fluorescent image was digitised by a PC VISION PC PLUS image analysis system. Using this system the image was digitised to pixels of varying gray level (gray levels range from zero to 255 with zero being black and 255 being white). The data were then converted to a histogram showing the number of pixels of each gray level within the image. From the histogram data, an average gray level was calculated for each image. This gray level was expressed as fluorescent intensity units and provides an index of macromolecular leakage in the area of interest (Schuschke *et al.*, 1989).

At the end of the *in vivo* experimentation, the size and wet weight of the tumour were recorded and specimens were taken for light and electron microscopy. Light microscopy specimens were fixed in buffered formaldehyde, dehydrated through graded alcohols and mounted in paraffin blocks. The paraffin blocks were then step sectioned at 5 μm intervals, placed on glass slides, and stained with routine hematoxylin and eosin. The angiogenic response was assessed semi-quantitatively by determining vascular density per high power field in the area of maximum vascular density (Brem *et al.*, 1972).

For electron microscopy, specimens (approximately 1 $mm^3$) were fixed in Karnovsky's fixative (paraformaldehyde/glutaraldehyde) in 0.2 N cacodylate buffer for 2 h, rinsed in buffer, post-fixed in osmium tetroxide in 0.144 N cacodylate buffer for 1 h followed by a buffer rinse. Specimens were subsequently dehydrated in an ascending graded series of ethanol followed by propylene oxide. Tissues were then immersed in propylene oxide/Epon 812-Araldite, first in a 1:1 mixture for 1 h followed by a 1:3 ratio for 4 h or overnight under vacuum. All tissues were then placed in 100% Epon 812-Araldite for 1 h, followed by embedding in the same epoxy resin. The resin tissue blocks were polymerised for 36 h, trimmed, and thick and thin sectioned on an ultramicrotome. Thick sections were mounted on glass slides, stained with toluidine blue, and viewed in a Nikon Microphot FX light microscope. Thin sections (70–80 nm) were collected on copper grids, stained with alcoholic uranyl acetate and aqueous lead citrate, and examined and photographed in a JEOL 100S transmission electron microscope.

The median lobe of the liver was removed and frozen for analysis of copper content. Specimens were subsequently freeze dried and digested in nitric acid and hydrogen peroxide (Nielsen *et al.*, 1982). Copper analysis was performed using a Jarrell-Ash Model 1140 inductively coupled plasma emission spectrometer.

Statistical analysis was by analysis of variance (ANOVA) followed by Tukey's studentised range test if an interaction between variables was revealed. Differences were considered significant at $P < 0.05$.

### Results

Diet analysis indicated that the CuD diet contained 0.38–0.55 μg Cu $g^{-1}$ diet and that the CuA diet contained 5.23–5.60 μg Cu $g^{-1}$ diet. The CuD diet caused a significantly lower liver copper content compared to the CuA contol group at all times tested (Figure 1). However, an extended time on the copper deficient diet did not significantly alter liver copper content from that seen at 5 days. Dietary copper deficiency did not cause changes in body weight, heart rate, or blood pressure (Table II).

*In vivo* experimentation did not demonstrate a difference in spontaneous macromolecular leakage from the tumour vasculature between the groups. Interstitial gray level within 1 min after FITC-BSA injection was not different between CuA and CuD groups (Figure 2) in either the 5 or 10-day groups. There was also no difference in gray level between groups 20 min after FITC-BSA injection. Analysis of the correlation between copper content and macromolecular leakage by linear regression gave a correlation coefficient of 0.54 which was not statistically significant.

Dietary copper deficiency did not to alter the rate of growth of the tumour at any of the times tested (Figure 3). There was also no difference in the vascular density during the development of the tumour (Figure 4).

Electron microscopy failed to demonstrate ultrastructural differences in tumour cells or endothelial cells between the CuA and CuD groups. The tumour cells within the lacunae had the typical morphology and associated circumferential extracellular matrix (ECM) which characterises chondrocytes and cartilaginous matrices. Associated with the ECM were fine non-striated Type II collagenous fibrils and scattered glycosaminoglycan matrix granules which typify the ECM associated with cartilage. The interface between endothelial cells was normal with tight junctions and interdigitating plasmalemmas. There was no demonstrable disruption of junctional complexes or significant thickening of the underlying basement membrane identified in the endothelial cells of tumour blood vessels.

### Discussion

Most studies involving the role of copper in angiogenesis have used the rabbit cornea as a model for experimentation. Results from experimentation with the cornea demonstrate that tissue copper levels increase prior to angiogenesis (Ziche *et al.*, 1982). Copper was previously shown to complex with fibroblast growth factor (FGF) (Shing, 1988), heparin, and ceruloplasmin (Raju *et al.*, 1984) thus making these agents angiogenic. Stimulation of vascularisation (Parke *et al.*, 1988)

**Figure 1** Hepatic copper concentration of animals fed either a diet adequate for copper (5 ppm) or deficient for copper (0 ppm). The diets were started 7 days before tumour implantation and the concentrations were quantitated 5, 10, or 20 days after the implantation. All values are X ± s.e. Two-way ANOVA indicated an interaction between diet and time ($P<0.02$) with Tukey's studentised range test showing a significant effect of diet at all times ($P<0.05$).

**Figure 2** Perivascular interstitial fluorescent intensity as an index of tumour venule macromolecular leakage. Measurements were made immediately after injection of FITC-BSA injection and again 20 min later. All values are X ± s.e. Three-way ANOVe showed no effect of diet, day or minute on fluorescent intensity (all $P$ values $>0.2$).

**Figure 3** Tumour weight as an index of growth 5, 10, and 20 days after tumour implantation. All values are X ± s.e. Two-way ANOVA indicated an effect of time ($P<0.003$) but no effect of diet ($P<0.9$) on tumour weight.

**Figure 4** Tumour vascular density as an index of angiogenesis 5, 10, and 20 days after tumour implantation. All values are X ± s.e. Two-way ANOVA indicated no effect of time or diet on vascular density ($P<0.2$).

**Table II** Characteristics of copper-adequate (CuA) and copper-deficient (CUD) animals

| | 5 day | | 10 day | | 20 day | |
|---|---|---|---|---|---|---|
| *Variable* | *CuA* | *CuD* | *CuA* | *CuD* | *CuA* | *CuD* |
| n size | 6 | 5 | 5 | 5 | 7 | 8 |
| Body Weight (grams) | 165 ± 4 | 162 ± 7 | 210 ± 7 | 215 ± 14 | 289 ± 13 | 262 ± 10 |
| Heart Rate (BPM) | 459 ± 13 | 445 ± 20 | 467 ± 25 | 466 ± 27 | 443 ± 21 | 429 ± 12 |
| Mean Blood Pressure (mmHg) | 124 ± 3 | 124 ± 5 | 130 ± 5 | 125 ± 5 | 124 ± 5 | 121 ± 3 |

All values are mean ± s.e.

and augmentation of endothelial locomotion *in vivo* (McAuslan & Reilly, 1980) are also copper-dependent.

Recently, evidence for inhibition of angiogenesis and tumour growth by copper depletion was presented. These studies included several types of human brain tumours implanted in rabbit cornea (Alpern-Elran & Brem, 1985), 9L gliosarcoma in rat brain (Brem *et al.*, 1990*a* and *b*), and $VX_2$ carcinoma in rabbit brain (Brem *et al.*, 1990*a*). In these studies, the animals were made copper deficient by dietary restriction of copper intake and the addition of the copper chelator penicillamine.

In contrast to these previous studies, we now report that a significant, diet-induced copper deficiency (Figure 1) does not alter tumour growth or angiogenesis in striated muscle. Tumour weight (Figure 3), microvascular density (Figure 4), and morphology were not influenced by dietary copper. There were also no differences in body weight, blood pressure, or heart rate in the CuD animals compared to controls (Table II).

In addition to tumour and blood vessel growth parameters there was no physiologic (Figure 2) evidence for a difference in tumour vasculature between CuA and CuD animals. Vascular integrity as assessed by macromolecular leakage was not different between the tumours of the CuA and CuD animals. While some tumours show an enhanced leakage with time, the chondrosarcoma was previously shown to have a relatively intact and 'non-leaky' vasculature (Heuser & Miller, 1986). In this study, copper deficiency did not alter the leakage characteristics of this tumour model (Figure 2).

There are several possible reasons for the differences seen in the present data compared to prior work with copper deficiency. The current study was done in animals made copper deficient by restriction of dietary intake of copper but without the addition of penicillamine. Since penicillamine has been shown to inhibit angiogenesis (Matsubara *et al.*, 1989) independent of its role as a copper chelator, it may have a role in the restriction of angiogenesis and tumour growth that has been attributed to copper deficiency.

Susceptibility to copper-deficiency may be tumour-specific. Tumour angiogenesis was previously shown to be dependent on the type of tumour implanted (Alpern-Elran & Brem, 1985). Their study demonstrated the inhibition of angiogenesis in some but not all human brain tumours implanted in the rabbit cornea of copper-depleted, penicillamine-treated rabbits.

Tumour angiogenesis also was shown to be host tissue-dependent. Brem *et al.* (1990*a*) reported that copper depletion and penicillamine treatment failed to inhibit tumour growth and the vascularisation of the $VX_2$ carcinoma in the rabbit thigh muscle while inhibition of tumour growth was present in the brain. This finding combined with ours may indicate that angiogenesis is copper-dependent in the CNS but not elsewhere.

Differences in the duration of copper deficiency prior to tumour implantation between the present study and previous work (1 *vs* 6 weeks) may be another possible explanation for the difference in reported results. Farquharson *et al.* (1989) proposed that tissue-specific cuproenzymes exist with different copper requirements for activity. There is also evidence for organ-specific uptake of copper (Pickart, 1983). This suggests that there is varying susceptibility of copper-dependent enzymes to copper deficiency. However, copper deficiency of the duration and level achieved in the present study has been previously shown to cause both biochemical and physiological alterations.

Endothelium-dependent and -independent vasoactive responses are significantly reduced after periods ranging from 17 to 27 days on the same copper deficient diet (Schuschke *et al.*, 1992). In addition, the same diet, within 1 week, produced a significant depression of plasma copper and liver copper concentration and reduced Cu,Zn-superoxide dismutase activity and within 14 days cytochrome c oxidase activity was depressed, iron status was reduced and there was significant anaemia. Within 21 days on the copper deficient diet there was significant cardiac hypertrophy (Johnson & Saari, 1991). Thus, while 7–21 days of this copper deficient diet has produced considerable alterations of normal tissues, there were no discernible effects on the tumour microcirculation in this study.

Based on results from the current study and other published reports, it appears that copper has a differential role in neovascularisation and tumour growth. The data suggest that the involvement of copper is either tumour-specific or host tissue-specific and it is likely that the interaction of the two tissues determine the final angiogenic and growth characteristics.

This research was supported by The Center for Applied Microcirculatory Research, University of Louisville, Louisville, Kentucky.

### References

ALPERN-ELRAN, H. & BREM, S. (1985). Angiogenesis in human brain tumors: inhibition by copper depletion. *Surg. Forum.*, **36**, 498–500.

BREM, S.B., COTRAN, R. & FOLKMAN, J. (1972). Tumor angiogenesis: a quantitative method for histologic grading. *J. Natl Cancer Inst.*, **48**, 347–356.

BREM, S.B., ZAGZAG, D., TSANACLIS, A.M.C., GATELY, S., ELKOUBY, M.-P. & BRIEN, S.E. (1990*a*). Inhibition of angiogenesis and tumor growth in the brain. *Am. J. Pathol.*, **137**, 1121–1142.

BREM, S.B., TSARACLIS, A.M.C. & ZAGZAG, D. (1990*b*). Anticopper treatment inhibits pseudopodial protrusion and the invasive spread of 9L gliosarcoma cells in the rat brain. *Neurosurgery*, **26**, 391–396.

FARQUHARSON, C., DUNCAN, A. & ROBBINS, S.P. (1989). The effects of copper deficiency on pyridinium crosslinks of mature collagen in the rat skeleton and cardiovascular system. *Proc. Soc. Exp. Biol. Med.*, **192**, 166–171.

FOLKMAN, J. & KLAGSBRUN, M. (1987). Angiogenic factors. *Science*, **235**, 442–447.

FOLKMAN, J. (1990). What is the evidence that tumors are angiogenesis dependent? *JNCI*,**82**,4–6.

HEUSER, L.S. & MILLER, F.N. (1986). Differential macromolecular leakage from the vasculature of tumors. *Cancer*, **57**, 461–464.

JOHNSON, W.T. & SAARI, J.T. (1991). Temporal changes in heart size, hematocrit and erythrocyte membrane protein in copper-deficient rats. *Nutr. Res.*, **11**, 1403–1414.

MATSUBARA, T., SAURA, R., HIROHATA, K. & ZIFF, M. (1989). Inhibition of human endothelial cell proliferation in vitro and neovascularisation *in vivo* by D-penicillamine. *J. Clin. Invest.*, **83**, 158-167.

MCAUSLAN, B.R. & REILLY, W. (1980). Endothelial cell phagokinesis in response to specific metal ions. *Exp. Cell Res.*, **130**, 147-157.

NIELSEN, F.H., ZIMMERMAN, T.J. & SHULER, T.R. (1982). Interactions among nickel, copper, and iron in rats. Liver and plasma contents of lipids and trace elements. *Biol. Trace Elem. Res.*, **4**, 125-143.

PARKE, A., BHATTARCHERJEE, P., PALMER, R.M. & LAZARUS, N.R. (1988). Characterization and quantification of copper sulfate-induced vascularization of the rabbit cornea. *Am. J. Path.*, **130**, 173-178.

PICKART, L. (1983). The biologic effects and mechanism of action of the plasma tripeptide glycyl-1-histadyl-1-lysine. *Lymphokines*, **8**, 425-446.

RAJU, K.S., ALESSANDRI, G. & GULLINO, P.M. (1984). Characterization of a chemoattractant for endothelium induced by angiogenesis effectors. *Cancer Res.*, **44**, 1579.

REED, M.W.R., WIEMAN, T.J., SCHUSCHKE, D.A., TSENG, M.T. & MILLER, F.N. (1989). A comparison of the effects of photodynamic therapy on normal and tumor blood vessels in the rat microcirculation. *Radiation Res.*, **119**, 542-552.

REPORT OF THE AMERICAN INSTITUTE FOR NUTRITION AD HOC COMMITTEE ON STANDARDS FOR NUTRITIONAL STUDIES. (1977). *J. Nutr.*, **107**, 1340-1348.

SCHUSCHKE, D.A., SAARI, J.T., ACKERMANN, D.M. & MILLER, F.N. (1989). Microvascular responses in copper-deficient rats. *Am. J. Physiol.*, **257**, H1607-H1612.

SCHUSCHKE, D.A., REED, M.W.R., SAARI, J.T. & MILLER, F.N. Differential effects of copper deficiency on vasodilation in the rat cremaster muscle microcirculation. *J. Nutr.* (in press).

SHING, Y. (1988). Heparin-Copper biaffinity chromatography of Fibroblast growth factors. *J. Biol. Chem.*, **263**, 9059-9062.

ZAGZAG, D., GOLDENBERG, M. & BREM, S. (1988). Neovascularisation and tumor growth in the rabbit brain. A model for experimental studies of angiogenesis and the blood brain barrier. *Am. J. Pathol.*, **131**, 361-371.

ZICHE, M., JONES, J. & GULLINO, P.M. (1982). Role of Prostaglandin E, and Copper in Angiogenesis. *JNCI*, **69**, 475-482.